# Quantifying the Risk to Health Care Workers of Cough as an Aerosol Generating Event in an Ambulance Setting: A Research Report

**DOI:** 10.1017/S1049023X22000917

**Published:** 2022-08

**Authors:** Dale A. Gedge, Robert P. Chilcott, Julia Williams

**Affiliations:** 1. University of Hertfordshire, School of Health and Social Work, Hatfield, Hertfordshire, United Kingdom; 2. Norfolk and Norwich University Hospital NHS Foundation Trust, Norwich, Norfolk, United Kingdom; 3. University of Hertfordshire, Toxicology Research Group, Hatfield, Hertfordshire, United Kingdom

**Keywords:** aerosol, cough, COVID-19, prehospital, SARS-CoV-2

## Abstract

**Introduction and Objective:**

United Kingdom Health Security Agency (UKHSA) guidance related to mask use for health care workers in a non-aerosol generating procedure (AGP) setting has remained as Level 2 water repellent paper mask (surgical mask) only. Energetic respiratory events, such as coughing, can generate vast numbers of droplets and aerosols. Coughing, considered to be a non-AGP event, frequently occurs in the relatively small, confined space of an ambulance (∼25 m^3^). The report seeks to explore whether existing research can provide an indication of the risk to ambulance staff, via aerosol transmission, of an acute respiratory infection (ARI) during a coughing event within the clinical setting of an ambulance.

**Methods:**

International bibliographic databases were searched (CINAHL Plus, SCOPUS, PubMed, and CENTRAL) using appropriate search strings and a combination of relevant medical subject headings with appropriate truncation. Methodological filters were not applied. Papers without an English language abstract were excluded from the review. Grey literature was sought by searching specialist databases OpenGrey and GreyNet, as well as key organizations’ websites. The initial search identified 2,405 articles. Following screening, along with forward and backward citation of key papers identified within the literature search, 36 papers were deemed eligible for the scoping review.

**Discussion:**

Attempts to replicate a clinical environment to investigate the risk of transmission of airborne viruses to health care workers during a coughing event provided evidence for the generation of respirable aerosol particles and thus potential transmission of pathogens. In cases of severe acute respiratory syndrome coronavirus-2 (SARS-CoV-2), potential to infect versus true airborne transmission is a debate that continues, but there is general consensus that a large variation of cough characteristics and aerosol generation amongst individuals exists. Studies widely endorsed face masks as a source control device, but there were conflicting views about the impact of mask leakage.

**Conclusion:**

Further research is required to provide clarity of the risk to health care workers when caring for a coughing patient in the confined clinical ambulance setting and to provide an evidence base to assist in the determination of appropriate respiratory protective equipment (RPE).

## Introduction

Given that severe acute respiratory syndrome coronavirus-2 (SARS-CoV-2) is capable of airborne transmission,^1–4^ adequate respiratory protective measures for health care workers are paramount. The clinical area in the back of an ambulance represents a unique environment for health care workers where ambulance staff may spend a prolonged period in this relatively small (∼25 m^3^), enclosed area with patients. The United Kingdom Health Security Agency (UKHSA; London, UK), formerly known as Public Health England (PHE), have recommended that ambulance personnel should carry out a “dynamic risk assessment” when attending suspected or confirmed coronavirus disease-19 (COVID-19) patients.^5^ Additionally, in September 2021, the concept of applying a “hierarchy of controls” to guide personal protective equipment choice was introduced,^6^ an approach ordinarily used to manage exposure to occupational hazards. Whilst the classification of respiratory protective equipment (RPE) to be worn in environments involving aerosol generating procedures (AGPs) has been outlined by UKHSA as a Level 3 filtering facepiece (FFP3) mask or a respirator/hood,^6^ the general guidance related to mask use for health care workers in non-AGP settings has remained as a Level 2 fluid-resistant surgical mask only (FRSM Type IIR).

There is a perceived importance of events labelled as AGPs in the transmission of viruses and other infectious agents in clinical settings, but the quantitative evidence to support this is lacking.^7–9^ Guidelines relating to AGPs^10^ are based on a systematic review^11^ with conclusions drawn from retrospective cohort studies that were all deemed to be of very low-quality.^12^ It is this evidence, where crucially aerosols were not measured, that has afforded AGPs their special status of an event considered to increase the risk of transmission of an airborne contagion.^13^

Studies have consistently found that traditional AGPs pose no greater risk than talking or breathing,^14^ whilst energetic respiratory events, such as coughing, can generate vastly increased numbers of droplets and aerosols.^15^ Significantly, recent studies have shown that a cough produces considerably more aerosol particles than numerous defined AGPs^2^ and yet cough is not classified as being aerosol generating by public health organizations. The UKHSA released updated guidance in January 2022 recommending that health care workers wear FFP3 masks when “caring for patients with a suspected or confirmed infection spread by the airborne route,”^16^ but with asymptomatic spread of SARS-CoV-2 widely accepted,^17–19^ this guidance does not go far enough to protect health care workers and reduce disease spread. Therefore, establishing whether a cough from an infected individual within the prehospital environment poses a significant risk to ambulance personnel will alleviate anxiety amongst this staff group and potentially shape future public health guidelines.

This report will outline themes within existing research that will contribute to a better understanding of the risks to health care workers from aerosol emissions produced by a coughing event within the ambulance setting.

## Methodology

**Figure 1.**
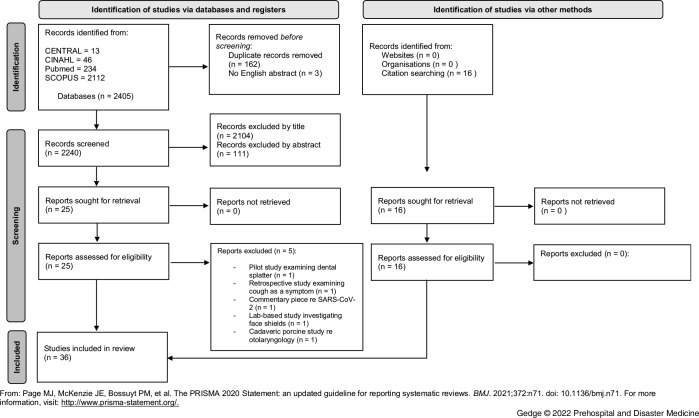
PRISMA 2020 Flow Diagram Outlining Search Results and Screening Process of Records Identified by the Literature Search.

The research question posed was “Is there a risk to the health care worker, via aerosol transmission, of an acute respiratory infection (ARI) during a coughing event whilst providing care for a patient with an ARI in an ambulance?” The following bibliographic databases were searched: CINAHL Plus (EBSCO Information Services; Ipswich, Massachusetts USA); SCOPUS (Elsevier; Amsterdam, Netherlands); PubMed (National Center for Biotechnology Information, National Institutes of Health; Bethesda, Maryland USA); and CENTRAL (The Cochrane Collaboration; London, United Kingdom). Grey literature was sought by searching specialist databases (OpenGrey [INIST-CNRS – Institut de l’Information Scientifique et Technique; Paris, France] and GreyNet [GreyNet International; Amsterdam, The Netherlands]) as well as key organizations’ websites: World Health Organization (WHO; Geneva, Switzerland), UKHSA, PHE, Resuscitation Council UK (London, UK), European Resuscitation Council (Niel, Belgium), Association of Ambulance Chief Executives (London, UK), International Liaison Committee on Resuscitation, and New and Emerging Respiratory Virus Threats Advisory Group. Methodological filters were not applied. Papers without an English language abstract were excluded from the review (n = 3). This initial search identified 2,405 articles with the breakdown per database detailed via a PRISMA flow diagram in Figure 1. One-hundred sixty-two duplicate publications were removed prior to screening. A total of 2,240 articles were initially screened by title with 2,104 articles removed, and then by abstract seeing 111 articles removed. The 25 remaining articles were assessed for eligibility via full-text review and consequently 20 papers were deemed eligible to be included in the scoping review. Forward and backward citation of key papers identified a further 16 articles that were appropriate for inclusion. The shortlist of 36 papers included in this review form a mixture of simulation studies (n = 16), modelling studies (10), case-control studies (2), literature reviews (2), general reviews (4), and commentary/discussion pieces (2).

## Discussion

### Cough in Simulated Clinical Settings

Simulation studies have tended to utilize either human volunteers or artificial simulators as the source of cough during experiments. The most applicable study identified from the literature search was performed in a chamber (to model a medical examination room) with a cough simulator used to generate an aerosol-laden cough and aerosol particle counters located at different positions within the room.^20^ With the study specifically focused on the aspect of infectious bioaerosols dispersed by patients in a health care environment and the risk to health care workers, aerosol particles with diameters of 0.3µm to 7.5µm were evaluated with results showing that cough-generated aerosol particles became rapidly dispersed throughout the room after just five minutes. As with any cough-simulator, a limitation of using machinery as the cough source is the inability to replicate the impact of buoyancy.^20^ The naturally heated human cough plume is usually warmer than the ambient air, hence its buoyancy, and although this may not have a significant impact on larger particles, it is likely to have a significant effect on smaller particle sizes.^21^ Additionally, cough simulators are unable to replicate the same real-world mechanisms of aerosol generation – primarily being shear stress as airflow meets the mucous membrane, vibration between structures in close proximity, and bronchial fluid burst on terminal airway reopening.^22^

A methodologically similar study to Lindsley, et al^20^ investigating the spread of the influenza pathogen during coughing concluded that aerosol transmission likely plays a role in the spread of influenza.^23^ Whether the results of studies looking at a specific pathogen can be generically applied to the airborne transmission of other infections is debatable, but Noti, et al’s^23^ study attempts to quantify infectivity from a coughing event which is relevant to other ARIs. Studies attempting to provide clarity regarding infectivity report the presence of airborne ribonucleic acid when reviewing both influenza and coronaviruses, but they rarely found viable viruses in the air.^24^ The SARS-CoV-2 virus has been detected in the air with a half-life of just over one hour,^1^ and this has numerous citations within the evidence base as proof of “viable” virus. However, Van Doremalen, et al’s^1^ study was laboratory based with an aerosolized environment created in a Goldberg drum, so it has a significant limitation of not being representative of real-world data. Following initial ambiguity, the UK government now recognizes that SARS-CoV-2 is transmitted via the airborne route,^25^ and the research broadly supports this concept.^12^

### Surgical Mask as a Source Control Device during Cough

Face mask efficacy is the primary focus of the human volunteer studies. Air flow leakage is an aspect that is often also reported during these studies where face masks are used as a source control device (ie, being worn by the source of the cough). Of the studies using human volunteers, a significant proportion used the Schlieren technique – a well-established method to visualize the flows of gases and liquids by use of differences in light refraction.^26^ However, this technique does not provide data on aerosol size, concentration, or mass distribution, so it is limited in inferring risk relating to aerosol transmission. The evidence shows considerable lateral air leakage around a surgical mask,^27–30^ with differing assertions on whether this effect redirects the jet-stream to a less harmful direction^27^ or should be considered as a major hazard for those in the vicinity of the cough.^28^ Researchers agree that a surgical mask is effective as a source control device but there is discrepancy in the degree of effect with a surgical mask recorded as blocking anywhere between 59%^30^ and ∼90%^2,31^ of aerosols produced by coughing. It is noteworthy that the study reporting poorer efficiency used a manikin head and cough simulator to test mask performance. With the UK guidance relating to the COVID-19 pandemic encouraging patients to wear a face mask during transportation in an ambulance,^6^ the element of face mask leakage is pertinent when weighing up risk to health care workers in a confined clinical setting such as an ambulance. Significantly, the evidence shows that loose-fitting face masks do not effectively prevent aerosol emissions contaminating the surrounding environment.^32^

### Cough Variation Amongst Individuals

Mathematical modelling studies are increasingly viewed as useful tools in clinical research, with the tendency being to use modelling when systematic reviews fail to adequately answer research questions. The results of modelling studies can be considered indicative with findings often determined by the validity of the primary data applied. The key parameter applied to modelling studies, and that which differs amongst the evidence, is the exhaled microdroplet/aerosol particle distribution and estimated viral copies produced during a cough. Variation amongst individuals has resulted in a “low” and “high” emitter range often being adopted with one modelling study finding that coughing emissions ranged between 0.000277 copies/cm^3^ (low emitter) to 36,030 copies/cm³ (high emitter) with the PM10 (particle size below 10µm) accounting for approximately one-half of these values.^33^ The aerosol number produced by a single cough has a range of between 900 to 300,000 particles when measuring aerosols between 0.35-10µm.^34^ The SARS-CoV-2 virus is thought to be 60-140nm in size^35^ and the virion particles have an affinity to attach to larger particles in the 0.3-10µm range,^36,37^ so it is entirely plausible that the virus could be contained within particles of the size range associated with aerosols. Riediker and Tsai’s^33^ study used research based on healthy individuals^38^ to form their modelling of microdroplet dispersal and application of previous research in this way highlights a significant flaw that can be directed towards modelling studies as infected individuals are thought to produce a higher concentration of aerosol.^2,34^

### Aerosol Emissions from Coughing whilst Infected

A case-control study found that particles per cough in infected (influenza) versus non-infected participants were reported as 75,400 and 52,200, respectively.^34^ The study reported particle size distribution for one participant considered to be a high emitter, displaying a generic increase in all size ranges when infected. This type of analysis would be particularly useful for the SARS-CoV-2 pathogen due to the inhibitory impact the virus has on surfactant production caused by the virion binding to ACE2 receptor sites and subsequently infecting and damaging Type II alveolar cells.^39^ Surfactant acts to reduce alveolar surface tension and is known to increase aqueous elasticity.^40^ In application to the SARS-CoV-2 infection, bronchiole fluid film burst may occur more frequently and at smaller diameters generating a larger volume of aerosols in the lower particle size range.

Although noting some significant limitations relating to reporting bias, Hamilton, et al^14^ cites a similar theme regarding aerosol particle number concentration from coughing when infected with SARS-CoV-2 as to that highlighted for influenza by Lindsley, et al.^34^ Hamilton, et al^14^ recruited hospitalized COVID-19 patients as a case cohort (n = 8) alongside a control cohort (n = 25) of healthy volunteers. Using optical and aerodynamic particle sizers, the volunteers underwent protocolized procedures, including coughing. The environment in which the research was carried out also represented a significant limitation, differing between the groups due to logistical constraints with an ultra-clean laminar flow operating theatre used for healthy volunteers and a negative pressure ventilated room used for hospitalized volunteers. Nevertheless, the study concluded that aerosol number concentration was higher during cough for the infected volunteers.

## Limitations

The literature search has failed to identify research that answers the initial research question posed. Whilst this is not a limitation in itself, the report discusses themes identified only within research that underwent full-text review as part of a screening process related to the posed research question.

## Conclusion

There is no unequivocal evidence to determine if public health guidelines are adequate in reducing the risk of exposure to SARS-CoV-2 for ambulance staff in the presence of infected patients. A limited number of studies have attempted to model the risk of transmission of airborne viruses to health care workers during a coughing event, but not within the confines of an ambulance. On balance, current information suggests that face masks may reduce the risk of infection, but there remains uncertainty due to mask leakage and lateral jet flows created by loose fitting surgical masks. It is clear that further research to establish quantitative risks to health care workers from aerosol emissions during a coughing event in an ambulance setting is required. Future research should aim to provide an evidence base from which appropriate RPE levels for health care workers exposed to a coughing patient can be determined.
